# Frailty as a Predictor of In-Hospital Outcomes in Patients Undergoing Percutaneous Coronary Intervention for Chronic Total Occlusion

**DOI:** 10.3390/jcm14134745

**Published:** 2025-07-04

**Authors:** Lourdes Vicent, Rafael Salguero-Bodes, Roberto Martín-Asenjo, Carlos Diaz-Arocutipa

**Affiliations:** 1 Department of Cardiology, Hospital Universitario 12 de Octubre, Instituto de Investigación Sanitaria Hospital 12 de Octubre (imas12), 28041 Madrid, Spain; mlourdesvicent@gmail.com (L.V.); rafael.salguero@salud.madrid.org (R.S.-B.); robertomartinasenjo@gmail.com (R.M.-A.); 2 Centro de Investigación Biomédica en Red Enfermedades Cardiovasculares (CIBERCV), 28041 Madrid, Spain; 3 Facultad de Medicina, Universidad Complutense, 28041 Madrid, Spain; 4 Unidad de Revisiones Sistemáticas y Meta-Análisis (URSIGET), Vicerrectorado de Investigación, Universidad San Ignacio de Loyola, Lima 15024, Peru

**Keywords:** frailty, percutaneous coronary intervention, chronic total occlusion, coronary artery disease

## Abstract

**Background/Objectives**: Data on the prognostic value of frailty in patients undergoing percutaneous coronary intervention (PCI) for chronic total occlusion (CTO) is limited. This study aimed to evaluate the association between frailty and in-hospital complications in patients undergoing CTO-PCI. **Methods**: We conducted a retrospective cohort study using administrative data from the National Inpatient Sample (2016–2019). Frailty was assessed using the Hospital Frailty Risk Score (HFRS) and categorized into three groups: low risk (<5), intermediate risk (5–15), and high risk (>15). Logistic regression models were applied to estimate odds ratios (ORs) with 95% confidence intervals (CIs) for in-hospital complications. **Results**: A total of 46,695 patients undergoing CTO-PCI were included. In the adjusted models, patients at high risk of frailty had higher odds of in-hospital mortality (OR 9.51, 95% CI 3.49–26.00), blood transfusion (OR 4.78, 95% CI 1.72–13.20), pericardial complication (OR 16.0, 95% CI 4.85–52.90), and renal replacement therapy (OR 3.83, 95% CI 1.22–12.00) compared to the low-risk group. Intermediate-risk patients also experienced higher odds of most outcomes. **Conclusions**: Frailty was a significant predictor of in-hospital complications in patients undergoing PCI for CTO. Incorporating frailty assessment into routine clinical practice could enhance risk stratification and enable tailored care strategies for this high-risk population.

## 1. Introduction

Chronic total occlusion (CTO) of the coronary arteries is one of the most complex and technically challenging forms of coronary artery disease. It is characterized by total occlusion of a coronary artery for more than three months and is associated with impaired myocardial perfusion, reduced quality of life, and an increased risk of adverse cardiovascular events [1]. In recent years, percutaneous coronary intervention (PCI) has gained traction as a revascularization strategy for CTO, demonstrating potential benefits in terms of angina relief, improvement in left ventricular function, and quality of life, particularly in appropriately selected patients [2,3].

Despite these advances, CTO-PCI procedures remain challenging due to the anatomical complexity of the lesions and the higher prevalence of comorbidities in this patient population [4]. Procedural success rates, though improving, still lag behind those of non-CTO-PCI, and complication rates—including perforation, tamponade, and contrast-induced nephropathy—are higher. Moreover, the patient population undergoing CTO-PCI is often older and burdened with multiple comorbidities, raising concerns about patient selection and periprocedural management.

Older adults with CTO often present with multiple cardiovascular and non-cardiovascular conditions that increase procedural risk and complicate recovery. Among these, frailty—a multidimensional syndrome reflecting diminished physiological reserve and increased vulnerability to stress—has emerged as a critical determinant of adverse outcomes in several cardiovascular interventions [5,6]. Frailty is a multidimensional construct encompassing physical, cognitive, and psychosocial domains. It is associated with decreased resilience to acute stress, slower recovery, and increased susceptibility to complications, including hospital-acquired infections, bleeding, and renal failure. In cardiovascular care, frailty has been linked to poor outcomes in patients undergoing transcatheter aortic valve replacement, cardiac surgery, and general PCI [7]. However, data specific to the prognostic value of frailty in the setting of CTO-PCI remain limited. Given the inherent complexity and high-risk nature of CTO procedures, a better understanding of the role of frailty in this context is needed to inform clinical decision-making and optimize periprocedural care. Patients with frailty often harbor additional risk factors such as polypharmacy, malnutrition, and cognitive impairment that may further complicate post-PCI recovery. Moreover, decision-making in frail patients is nuanced, with an imperative to balance procedural benefit against risk, patient values, and anticipated functional trajectories.

Understanding the relationship between frailty and CTO-PCI outcomes could also guide the development of personalized, multidisciplinary care strategies. For example, high frailty scores may prompt considerations such as delaying intervention for prehabilitation, adapting procedural techniques to minimize contrast or procedural time, or involving geriatricians in care planning. Additionally, knowledge of frailty burden can inform conversations about goals of care, especially in patients with borderline indications for revascularization.

The aim of this study was to evaluate the association between frailty and in-hospital outcomes in patients undergoing PCI for CTO using data from a large, nationally representative database. We hypothesized that frailty would be independently associated with worse in-hospital outcomes, including mortality and procedure-related complications.

## 2. Materials and Methods

### 2.1. Study Design and Data Source

We conducted a retrospective cohort study utilizing data from the National Inpatient Sample, the largest publicly available inpatient healthcare database in the United States. Developed as part of the Healthcare Cost and Utilization Project by the Agency for Healthcare Research and Quality, the National Inpatient Sample database includes data from approximately 7 million hospital stays each year and provides national estimates using stratified sampling techniques. The database contains patient demographics, diagnoses, procedures, hospital characteristics, and outcomes, making it ideal for health services and outcomes research.

The study period spanned from 1 January 2016 to 31 December 2019. We selected this timeframe to align with the adoption of the ICD-10-CM/PCS coding system in the US hospital system. This uniform coding environment ensures consistency in the identification of diagnoses and procedures across the study population. To enhance data reliability, we adhered to best practices for handling complex survey design by applying discharge-level weights, strata, and cluster design variables provided by the National Inpatient Sample database. These adjustments ensure the derivation of nationally representative estimates and account for sampling variability across hospitals.

This study was conducted in accordance with the ethical principles of the Declaration of Helsinki for research involving human subjects. Informed consent was not required because the analysis used the National Inpatient Sample database, a publicly available and de-identified administrative database maintained by the Healthcare Cost and Utilization Project. The National Inpatient Sample database contains anonymized patient-level data and does not include personal identifiers. As such, the use of this dataset is considered exempt from institutional review board oversight, and it poses minimal risk to patient privacy.

### 2.2. Study Population

Adult patients (≥18 years of age) who underwent a PCI procedure for CTO between 2016 and 2019 were included, using International Classification of Diseases, Tenth Revision (ICD-10) codes (Table S1). Patients with a primary or secondary diagnosis of acute myocardial infarction were excluded. The ICD-10 code I25.82 to identify patients with CTO was used, as considered in previous studies [8,9].

### 2.3. Frailty

The Hospital Frailty Risk Score was used to assess frailty status in individual patients [10]. This tool estimates the risk of frailty among hospitalized patients by utilizing ICD-10 codes derived from administrative data. The Hospital Frailty Risk Score quantifies frailty risk using 109 diagnostic codes associated with comorbid conditions such as dementia, cognitive impairment, falls, pressure ulcers, urinary incontinence, and mobility limitations, among others. Each code is assigned a weighted value according to its predictive association with frailty-related outcomes. The final score stratifies patients into three categories of frailty risk, low (<5), intermediate (5–15), and high (>15), as used in the study by Gilbert et al. and other studies [10,11,12]. This classification enables researchers and clinicians to efficiently identify individuals at elevated risk for adverse outcomes without the need for direct clinical assessment, making it particularly useful in large-scale retrospective analyses and administrative database research. The Hospital Frailty Risk Score has been validated in multiple clinical settings and has demonstrated good predictive performance for adverse outcomes such as prolonged hospitalization, unplanned readmission, and mortality [13,14]. Its use is especially relevant in cardiovascular populations, where frailty is increasingly recognized as an important determinant of procedural risk and recovery trajectory. In the context of PCI for CTO, the Hospital Frailty Risk Score provides a practical and scalable method to capture frailty burden, facilitating the integration of this important geriatric construct into risk stratification and outcome prediction.

### 2.4. Outcomes

The primary outcome was in-hospital mortality, and secondary outcomes were vascular complication, blood transfusion, coronary perforation/dissection, pericardial complication, and renal replacement therapy. All outcomes were identified through ICD-10 diagnostic and procedural codes from administrative data, ensuring standardized and reproducible definitions across the study cohort. A complete list of codes used for outcome identification is provided in Table S1.

### 2.5. Covariates

Sociodemographic characteristics, including age, sex, race/ethnicity, household income estimated by zip code, type of admission, and comorbidities based on the Elixhauser Comorbidity Index, were collected. In addition, hospital-specific characteristics such as bed size of hospital, location of hospital, region of hospital, and ownership of hospital were also recorded.

### 2.6. Statistical Analysis

Categorical data were presented as frequencies and percentages, while continuous data were reported as medians (interquartile range [IQR]). Associations between categorical variables were evaluated using the chi-squared test with Rao & Scott’s second-order correction, and the Kruskal–Wallis rank sum test was applied for continuous variables. To examine the association between frailty (using low risk as the reference group) and all outcomes, logistic regression models were used to estimate odds ratios (ORs) with corresponding 95% confidence intervals (CIs). The following covariates were previously selected to be included in the adjusted models: age, sex, race/ethnicity, elective admission, comorbidity burden, primary expected payer, hospital bed size, and hospital location. As a sensitivity analysis, length of hospital stay was included as a covariate in the adjusted regression models. Multicollinearity among independent variables was assessed using the Variance Inflation Factor (VIF). A threshold of VIF > 5 was considered indicative of potential collinearity. Additionally, restricted cubic splines with four prespecified knots were used to assess nonlinear relationships between the frailty score (reference value: 5 points) and binary outcomes. All statistical analyses were performed using R software, version 4.3.2 (R Foundation for Statistical Computing, Vienna, Austria). A two-tailed *p*-value < 0.05 was considered statistically significant.

## 3. Results

**Figure 1:**
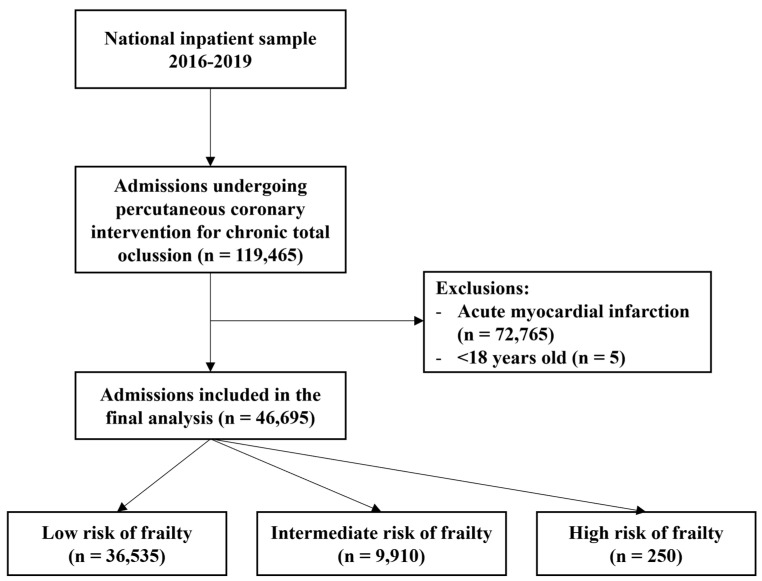
Flow diagram for the selection of study participants.

A total of 46,695 weighted hospital admissions for patients undergoing PCI for CTO were included in the analysis (Figure 1). According to the Hospital Frailty Risk Score, patients were classified as low (*n* = 36,535), intermediate (*n* = 9910), and high frailty risk (*n* = 250). Baseline demographic and clinical characteristics varied significantly across frailty categories. Median age increased from 67 years in the low-risk group to 73 years in the high-risk group (*p* < 0.001), and the proportion of female patients was higher among those with intermediate (32%) and high frailty (34%) compared to the low-risk group (25%) (*p* < 0.001). Most patients across all categories were of White race (71%), and admissions were more frequently elective among low-risk individuals (28%) compared to those in the intermediate (21%) and high-risk (24%) groups (*p* < 0.001). Medicare coverage was more prevalent in frailer patients, representing 61% of low-risk and 80% of high-risk admissions (*p* < 0.001). Full baseline characteristics are presented in Table 1.

The burden of comorbidities increased progressively with frailty. High-risk patients exhibited markedly higher rates of diabetes (72%), heart failure (62%), renal failure (54%), and atrial fibrillation (35%) compared to the low-risk group (47%, 39%, 18%, and 16%, respectively) (all *p* < 0.001). Prior stroke or TIA was also more common among frail patients, whereas previous myocardial infarction and PCI were more frequently documented in low-risk individuals. The Elixhauser Comorbidity Index rose significantly across frailty categories, with median scores of 3.0, 4.0, and 6.0 for low, intermediate, and high-risk patients, respectively (*p* < 0.001). Additionally, resource utilization increased with frailty: median hospital length of stay rose from 2.0 days in low-risk to 10.0 days in high-risk patients, and total hospital charges doubled across groups, from USD 76,337 to USD 146,231 (*p* < 0.001 for both comparisons).

**Figure 2:**
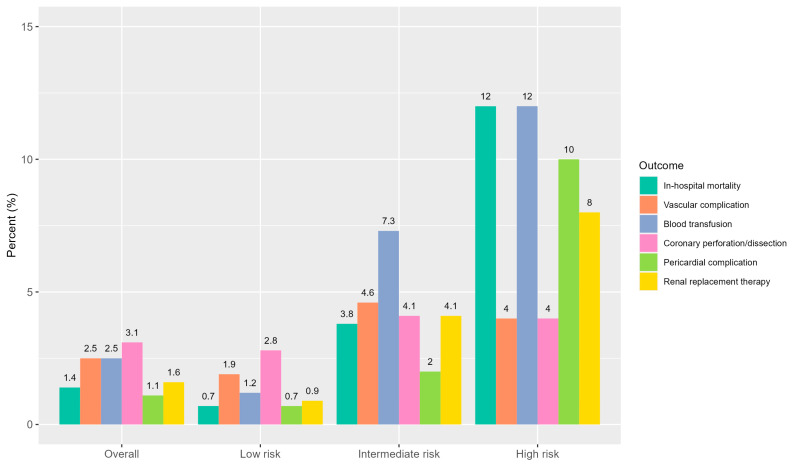
Primary and secondary outcomes according to frailty groups.

In-hospital adverse events were more common with increasing frailty severity (Figure 2). In-hospital mortality rates were 0.7%, 2.9%, and 12.0% across the low-, intermediate-, and high-risk groups, respectively (*p* < 0.001). The incidence of vascular complications, transfusion-requiring bleeding, renal replacement therapy, and pericardial complications also followed this gradient. For example, blood transfusions were administered in 2.7% of low-risk patients compared to 16.4% of high-risk patients. Pericardial complications occurred in 10.0% of high-risk patients versus 1.5% in the low-risk group (*p* < 0.001). Coronary perforation or dissection occurred more frequently in intermediate- and high-risk patients (*p* = 0.012).

In multivariable regression models adjusting for age, sex, race, comorbidities, and hospital characteristics, increasing frailty remained independently associated with worse clinical outcomes (Table 2). Compared with low-risk patients, those at intermediate frailty risk had significantly higher odds of in-hospital mortality (OR 3.40, 95% CI 2.26–5.11), vascular complications (OR 2.27, 95% CI 1.66–3.11), blood transfusion (OR 3.56, 95% CI 2.57–4.95), coronary perforation or dissection (OR 1.59, 95% CI 1.18–2.14), pericardial complications (OR 3.43, 95% CI 2.08–5.68), and renal replacement therapy (OR 1.90, 95% CI 1.29–2.81). High-risk frailty was associated with even greater odds of in-hospital mortality (OR 9.51, 95% CI 3.49–26.00), transfusion (OR 4.78, 95% CI 1.72–13.20), pericardial complications (OR 16.0, 95% CI 4.85–52.9), and renal replacement therapy (OR 3.83, 95% CI 1.22–12.0). However, associations with vascular complications and coronary perforation or dissection were no longer significant in this group after adjustment.

Frailty was also strongly associated with healthcare resource utilization. In adjusted models, intermediate-risk patients experienced a mean increase of 2.7 days in length of stay (95% CI 2.4–2.9) and USD 43,667 in additional hospital charges (95% CI 37,359–49,975). High-risk patients had an even greater increase of 7.0 days (95% CI 4.9–9.0) and USD 82,031 (95% CI 34,096–129,965) (all *p* < 0.001).

**Figure 3:**
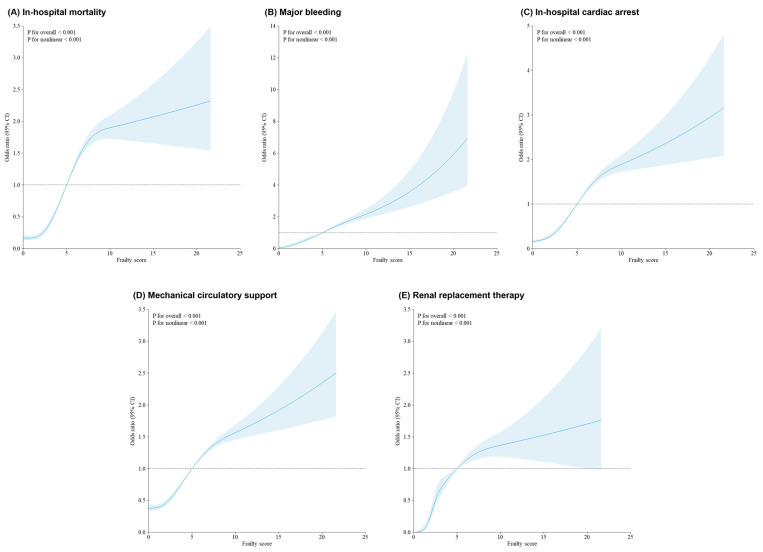
Association between frailty and all outcomes using a restricted cubic spline regression model. Graphs show the ORs for all outcomes according to frailty score as continuous data. The solid lines indicate the ORs and the gray region indicates the 95% CIs. Abbreviations: OR, odds ratio; CI, confidence interval.

Restricted cubic spline models demonstrated a nonlinear association between frailty score and the likelihood of adverse outcomes (Figure 3). Risk began rising steeply at a score of approximately 5 and continued to increase at higher frailty levels. Sensitivity analyses including length of stay as an additional covariate yielded consistent findings, supporting the robustness of the primary results (Table S2). Finally, multicollinearity among covariates included in regression models was assessed using the Variance Inflation Factor (VIF). All variables had VIF values below 2, indicating the absence of significant collinearity.

## 4. Discussion

In this large, nationally representative cohort of patients undergoing CTO-PCI, frailty—quantified using the Hospital Frailty Risk Score—was identified as a strong and independent predictor of multiple adverse in-hospital outcomes. Patients classified as frail, particularly those in the high-risk category, experienced significantly greater odds of mortality, major bleeding requiring transfusion, vascular and pericardial complications, coronary perforation or dissection, renal replacement therapy, longer hospital stays, and higher hospitalization costs. These associations remained robust even after multivariable adjustment for a wide array of demographic, clinical, and hospital-level characteristics, highlighting the prognostic value of frailty in this high-risk patient population.

A particularly notable observation was the nonlinear association between frailty and adverse outcomes, as evidenced by the restricted cubic spline analysis. Such findings are clinically meaningful, as they indicate that even moderate frailty may substantially compromise the patient’s physiological reserve and increase susceptibility to the procedural stressors of CTO-PCI. Frailty likely reflects a complex interaction between aging, comorbid burden, sarcopenia, nutritional deficiencies, systemic inflammation, and decreased homeostatic capacity—factors that may not be adequately captured by traditional risk scores [15].

CTO-PCI procedures are inherently complex, involving long procedural times, higher contrast use, and advanced techniques such as dissection/re-entry, retrograde approaches, and extensive balloon or stent deployment. These procedures require specialized operator expertise and are often performed in patients with multiple comorbidities, including diabetes, chronic kidney disease, and heart failure, which further increase the risk of complications [16,17,18]. Frail patients, already characterized by impaired physiological resilience, may be particularly vulnerable to these challenges. In our cohort, frail patients had a higher prevalence of comorbidities such as diabetes, chronic kidney disease, and heart failure—each independently associated with adverse procedural outcomes. The convergence of frailty and comorbidity may create a synergistic effect, amplifying vulnerability to complications such as acute kidney injury, tamponade, or bleeding [6]. For instance, the increased rates of pericardial complications and renal replacement therapy among frail patients likely reflect this compounded risk.

Our findings have important implications for clinical practice. Preprocedural frailty assessment can provide valuable information for risk stratification and decision-making. The Hospital Frailty Risk Score, based on ICD-10 codes, offers a scalable and validated approach for identifying frailty using routinely collected administrative data [19]. Compared to more subjective or time-consuming tools—such as gait speed, grip strength, or clinician gestalt—the HFRS can be implemented at scale and integrated into electronic health records to facilitate automated risk flagging [20]. Incorporating frailty assessment into routine workflow may help inform patient counseling, guide procedural planning, and support resource allocation, especially in centers where CTO-PCI is selectively performed due to its technical complexity and cost. It is important to note, however, that patients classified as highly frail accounted for only 0.5% of the study population, which limits the precision of the estimates for this subgroup. Although the observed associations were directionally consistent with those seen in the intermediate frailty group, the wide confidence intervals suggest that these findings should be interpreted with caution. Larger cohorts of highly frail patients will be required to validate these results.

Moreover, recognizing frailty before PCI may enable proactive periprocedural optimization strategies. Several studies have demonstrated the benefit of multidisciplinary care for frail cardiovascular patients, including prehabilitation programs, nutritional optimization, medication reconciliation, and early mobilization [21,22,23,24,25]. Such interventions could potentially mitigate the elevated risk observed in frail patients undergoing CTO-PCI. For example, minimizing contrast volume, utilizing radial access when feasible, employing intravascular imaging to limit procedural time, or staging complex interventions may help reduce complications in this population [5,26]. Additionally, frailty identification may prompt the involvement of geriatricians, palliative care teams, or case managers in cases where procedural benefit is uncertain or limited.

Our study also highlights the broader value of frailty as an integrative biomarker. Unlike isolated comorbidities, frailty captures the cumulative effect of multiple physiological deficits, many of which interact and potentiate each other. For instance, a frail patient with marginal renal function and borderline nutritional status may be far more vulnerable to procedural stress than a robust patient with the same comorbid diagnoses [23]. Thus, frailty may help clinicians better identify which patients are at the highest risk despite similar traditional risk profiles. This could be particularly useful in borderline or elective cases where the procedural indication is nuanced and the risks must be carefully balanced against expected benefits. However, the absence of procedural success data—such as lesion crossing or restoration of flow—limits the assessment of how frailty modifies the net clinical benefit of CTO-PCI. It is important to acknowledge that the present analysis did not include procedural success metrics, such as lesion crossing, restoration of TIMI flow, or procedural duration, due to limitations inherent to the administrative dataset. This represents a potential confounder, as failed, prolonged, or complicated procedures are known to increase the risk of adverse outcomes. It is plausible that patients with higher frailty scores may also present with more complex coronary anatomy or challenging vascular access, thereby increasing the likelihood of procedural failure. As a result, some of the observed associations between frailty and poor outcomes may be mediated by unmeasured procedural characteristics. Nevertheless, the persistence of strong associations after multivariable adjustment—including demographic, clinical, and hospital-level factors—suggests that frailty independently contributes to adverse in-hospital outcomes. Future prospective studies that incorporate anatomical complexity and procedural success data will be crucial to disentangle these interrelated pathways and refine risk prediction in patients undergoing CTO-PCI. While our analysis focused on adverse outcomes, future studies should include technical success and long-term benefit to fully inform risk–benefit assessments in frail patients.

The strengths of this study include the large sample size, the use of a nationally representative dataset, and the application of a validated and reproducible frailty assessment tool. The use of restricted cubic spline regression allowed for the exploration of nonlinear relationships between frailty and outcomes, offering deeper insight into risk thresholds and gradients. In addition, the rigorous adjustment for a wide range of confounders enhances the validity of our findings and reduces the likelihood that observed associations are due to unmeasured bias.

Nevertheless, several limitations must be acknowledged. First, the retrospective nature of the analysis using administrative data is subject to potential misclassification and coding inaccuracies. Frailty was not assessed directly but inferred from diagnostic codes using the Hospital Frailty Risk Score. Although this score has been validated in hospitalized patients, it may not capture all dimensions of frailty, particularly cognitive, emotional, and social vulnerabilities, which are increasingly recognized as important predictors of clinical outcomes. In addition, our outcome definition of all-cause in-hospital mortality does not allow differentiation between procedural and non-procedural causes of death. It is possible that some deaths among frail patients were related to factors such as infections, aspiration, or functional decline rather than direct procedural complications. While this limits our ability to define precise causal pathways, we opted for an all-cause definition to minimize misclassification and maintain consistency. Moreover, the observed associations between frailty and procedure-related complications—including bleeding, pericardial complications, and need for dialysis—suggest that at least part of the increased mortality risk is likely procedure-associated. Second, the National Inpatient Sample dataset does not contain information on long-term outcomes such as 30-day readmissions, 1-year survival, or health-related quality of life—important considerations when evaluating procedural benefit in frail patients. Third, and importantly, the National Inpatient Sample lacks detailed procedural data such as lesion complexity (e.g., J-CTO score), contrast volume, procedural success, or operator experience—factors known to influence CTO-PCI outcomes. While we adjusted for hospital characteristics such as teaching status, region, and bed size as partial proxies, the absence of direct procedural metrics limits the ability to fully account for procedural risk and technical variability. Future prospective studies that integrate both clinical and procedural information will be essential to confirm these findings and refine risk stratification. Although this database offers national representativeness and large-scale power, it does not include granular clinical details such as symptom burden or ischemia severity, which may influence the decision to perform CTO-PCI. It is also possible that frailty plays a role in procedural selection, whereby frail patients are offered revascularization only under specific clinical circumstances. Although this could introduce a degree of selection bias, we believe the consistency and strength of the observed associations—adjusted for a broad range of clinical and hospital-level factors—support the prognostic relevance of frailty in this setting. Prior studies from the National Cardiovascular Data Registry and other registries have shown that CTO-PCI outcomes are highly operator-dependent and vary significantly across centers [27,28,29,30]. Differences in institutional expertise, procedural planning, and postprocedural care may also influence outcomes but were not directly measurable in our study. While we attempted to account for some of these factors by adjusting for hospital characteristics (e.g., teaching status, bed size, region), these variables are imperfect proxies for operator skill and experience. Finally, although we adjusted for numerous patient-level and hospital-level variables, residual confounding may persist. For example, frailty may be associated with unmeasured variables such as poor nutrition, caregiver availability, or functional decline—factors that influence both treatment decisions and outcomes but are not captured in administrative data. Furthermore, there was no information on the medications administered in the hospital, such as anticoagulants, antiplatelet agents, or nephroprotective therapies. These treatments could influence outcomes such as bleeding, kidney injury, or mortality.

Future prospective studies are needed to validate these findings in clinical settings and to determine whether frailty-directed care strategies can improve outcomes in patients undergoing CTO-PCI. Trials evaluating prehabilitation, geriatric co-management, or tailored procedural strategies based on frailty status would be particularly valuable. Furthermore, expanding frailty assessments to include functional status, cognition, and psychosocial support may provide a more comprehensive framework for risk stratification and care planning in this vulnerable population.

To enhance the clinical applicability of our findings, we have included a summary table (Table 3) outlining in-hospital outcomes and potential clinical implications stratified by frailty group. This table consolidates key differences in short-term prognosis and provides a practical framework to support decision-making. For instance, while patients in the low-risk group generally experience favorable outcomes with standard CTO-PCI approaches, those at intermediate risk may benefit from targeted optimization strategies, such as renal protection and bleeding risk mitigation. In contrast, highly frail patients exhibit markedly increased rates of mortality, pericardial complications, and need for dialysis, underscoring the importance of frailty-informed procedural planning. For this subgroup, alternative strategies—such as staged procedures, less invasive techniques, or even conservative management—may be appropriate. This summary may aid clinicians in tailoring care based on frailty status, improving periprocedural safety, and optimizing resource allocation in this high-risk population.

## 5. Conclusions

Frailty was a significant determinant of in-hospital outcomes in patients undergoing CTO-PCI. Routine assessment of frailty using tools such as the Hospital Frailty Risk Score can provide valuable prognostic insights and inform individualized care strategies. These findings highlight the need for a paradigm shift towards frailty-informed care in interventional cardiology, particularly for high-risk populations such as those with CTO. By addressing frailty as a modifiable factor, clinicians can improve procedural safety, enhance recovery, and ultimately improve the quality of care for this vulnerable population.

## Figures and Tables

**Table 1:** Characteristics of admissions according to frailty groups.

Characteristic	Overall	Frailty Groups			*p*-Value
		Low Risk	Intermediate Risk	High Risk	
**Weighted admissions**	46,695	36,535	9910	250	
Age (years) *	68 (60–76)	67 (59–75)	71 (63–78)	73 (65–80)	<0.001
**Female sex**					<0.001
Male	34,405 (74%)	27,510 (75%)	6730 (68%)	165 (66%)	
Female	12,290 (26%)	9025 (25%)	3180 (32%)	85 (34%)	
**Race/ethnicity**					<0.001
Other	5425 (12%)	4500 (12%)	895 (9%)	30 (12%)	
White	33,175 (71%)	25,920 (71%)	7075 (71%)	180 (72%)	
Black	4295 (9%)	3180 (9%)	1095 (11%)	20 (8%)	
Hispanic	3800 (8%)	2935 (8%)	845 (9%)	20 (8%)	
**Elective admission**	12,215 (26%)	10,100 (28%)	2055 (21%)	60 (24%)	<0.001
**Household income**					0.049
Quartile 1	13,390 (29%)	10,265 (29%)	3060 (31%)	65 (27%)	
Quartile 2	11,920 (26%)	9215 (26%)	2635 (27%)	70 (29%)	
Quartile 3	11,015 (24%)	8820 (25%)	2135 (22%)	60 (25%)	
Quartile 4	9535 (21%)	7605 (21%)	1885 (19%)	45 (19%)	
**Hypertension**	41,875 (90%)	32,595 (89%)	9060 (91%)	220 (88%)	0.015
**Diabetes**	23,085 (49%)	17,005 (47%)	5900 (60%)	180 (72%)	<0.001
**Dyslipidemia**	37,805 (81%)	30,075 (82%)	7560 (76%)	170 (68%)	<0.001
**Congestive heart failure**	20,845 (45%)	14,145 (39%)	6545 (66%)	155 (62%)	<0.001
**Chronic pulmonary disease**	10,560 (23%)	7460 (20%)	3030 (31%)	70 (28%)	<0.001
**Atrial fibrillation**	9420 (20%)	6310 (17%)	3020 (30%)	90 (36%)	<0.001
**Valvular disease**	8275 (18%)	5805 (16%)	2435 (25%)	35 (14%)	<0.001
**Renal failure**	12,230 (26%)	6610 (18%)	5485 (55%)	135 (54%)	<0.001
**Previous myocardial infarction**	15,530 (33%)	12,260 (34%)	3230 (33%)	40 (16%)	0.025
**Previous stroke/TIA**	5280 (11%)	3235 (9%)	1955 (20%)	90 (36%)	<0.001
**Previous PCI**	14,530 (31%)	11,835 (32%)	2640 (27%)	55 (22%)	<0.001
**Previous CABG**	13,605 (29%)	11,115 (30%)	2430 (25%)	60 (24%)	<0.001
Elixhauser Comorbidity Index *	4.00 (2.00–5.00)	3.00 (2.00–5.00)	6.00 (4.00–7.00)	6.00 (5.00–8.00)	<0.001
**Expected insurance payer**					<0.001
Medicare	29,995 (64%)	22,320 (61%)	7475 (76%)	200 (80%)	
Medicaid	4020 (9%)	3355 (9%)	655 (7%)	10 (4%)	
Private	10,140 (22%)	8790 (24%)	1320 (13%)	30 (12%)	
Other	2485 (5%)	2025 (6%)	450 (5%)	10 (4%)	
**Bed size of hospital**					0.744
Small	6670 (14%)	5235 (14%)	1400 (14%)	35 (14%)	
Medium	11,900 (25%)	9190 (25%)	2645 (27%)	65 (26%)	
Large	28,125 (60%)	22,110 (61%)	5865 (59%)	150 (60%)	
**Location of hospital**					0.347
Rural	1650 (4%)	1340 (4%)	310 (3%)	0 (0%)	
Urban non-teaching	8845 (19%)	6840 (19%)	1945 (20%)	60 (24%)	
Urban teaching	36,200 (78%)	28,355 (78%)	7655 (77%)	190 (76%)	
**Region of hospital**					<0.001
Northeast	10,395 (22%)	8720 (24%)	1645 (17%)	30 (12%)	
Midwest	10,625 (23%)	7985 (22%)	2520 (25%)	120 (48%)	
South	17,490 (37%)	13,570 (37%)	3845 (39%)	75 (30%)	
West	8185 (18%)	6260 (17%)	1900 (19%)	25 (10%)	
**Ownership of hospital**					0.276
Government, nonfederal	3405 (7%)	2585 (7%)	795 (8%)	25 (10%)	
Private, non-profit	36,190 (78%)	28,310 (77%)	7675 (77%)	205 (82%)	
Private, investor-owned	7100 (15%)	5640 (15%)	1440 (15%)	20 (8%)	
**Transfer out indicator**					<0.001
Not a transfer	43,540 (93%)	35,240 (97%)	8200 (83%)	100 (40%)	
Different acute care hospital	510 (1%)	370 (1%)	135 (1%)	5 (2%)	
Another type of health facility	2605 (6%)	885 (2%)	1575 (16%)	145 (58%)	
Length of hospital stay (days) *	3.0 (1.0–5.0)	2.0 (1.0–4.0)	5.0 (3.0–9.0)	10.0 (5.0–16.5)	<0.001
Total charges (USD) *	83,169 (57,583–129,964)	76,337 (54,675–116,101)	116,749 (76,526–189,485)	146,231 (94,915–248,768)	<0.001
Frailty score *	2.10 (0.70–4.50)	1.50 (0.00–2.90)	7.00 (5.80–9.00)	16.90 (15.95–18.75)	<0.001

* Median (p25–p75).

**Table 2:** Univariable and multivariable model analyses between frailty groups and outcomes.

Outcomes	Crude Model			Adjusted Model *		
	**OR**	**95% CI**	***p*** **-Value**	**OR**	**95% CI**	***p*** **-Value**
**In-hospital mortality**						
Low risk of frailty	Ref.			Ref.		
Intermediate risk of frailty	5.82	4.05–8.37	<0.001	3.40	2.26–5.11	<0.001
High risk of frailty	20.2	8.22–49.5	<0.001	9.51	3.49–26.0	<0.001
**Vascular complication**						
Low risk of frailty	Ref.			Ref.		
Intermediate risk of frailty	2.46	1.88–3.21	<0.001	2.27	1.66–3.11	<0.001
High risk of frailty	2.10	0.51–8.74	0.307	1.49	0.35–6.31	0.588
**Blood transfusion**						
Low risk of frailty	Ref.			Ref.		
Intermediate risk of frailty	6.66	5.07–8.75	<0.001	3.56	2.57–4.95	<0.001
High risk of frailty	11.6	4.81–27.9	<0.001	4.78	1.72–13.2	0.003
**Coronary perforation/dissection**						
Low risk of frailty	Ref.			Ref.		
Intermediate risk of frailty	1.48	1.14–1.92	0.003	1.59	1.18–2.14	0.002
High risk of frailty	1.43	0.34–5.92	0.622	1.32	0.32–5.39	0.701
**Pericardial complication**						
Low risk of frailty	Ref.			Ref.		
Intermediate risk of frailty	2.77	1.83–4.18	<0.001	3.43	2.08–5.68	<0.001
High risk of frailty	14.9	5.70–39.1	<0.001	16.0	4.85–52.9	<0.001
**Renal replacement therapy**						
Low risk of frailty	Ref.			Ref.		
Intermediate risk of frailty	4.47	3.23–6.19	<0.001	1.90	1.29–2.81	0.001
High risk of frailty	9.12	3.20–26.0	<0.001	3.83	1.22–12.0	0.021
	**Mean difference**	**95% CI**	***p*** **-value**	**Mean difference**	**95% CI**	***p*** **-value**
**Length of hospital stay (days)**						
Low risk of frailty	Ref.			Ref.		
Intermediate risk of frailty	4.0	3.7–4.3	<0.001	2.7	2.4–2.9	<0.001
High risk of frailty	8.7	6.6–11	<0.001	7.0	4.9–9.0	<0.001
**Total charges (USD)**						
Low risk of frailty	Ref.			Ref.		
Intermediate risk of frailty	60–553	54,261–66,844	<0.001	43 -667	37,359–49,975	<0.001
High risk of frailty	104–1031	56,603–151,460	<0.001	82 -031	34,096–129,965	<0.001

OR, odds ratio; CI, confidence interval; USD, US dollars. * Adjusted for age, sex, race/ethnicity, elective admission, expected insurance payer, bed size of hospital, and location of hospital.

**Table 3:** Summary of clinical implications by frailty group.

Frailty Group	In-Hospital Outcomes	Clinical Implications
Low risk (<5 points)	Low mortality and complication rates	- Suitable candidates for standard CTO-PCI strategies. - Routine risk mitigation measures apply. - Less intensive postprocedural monitoring generally sufficient.
Intermediate risk (5–15 points)	Moderate increase in mortality, bleeding, renal complications	- Preprocedural risk stratification and optimization recommended. - Consider prehabilitation, renal protection, and bleeding risk management. - Multidisciplinary planning may enhance outcomes.
High risk (>15 points)	Highest rates of mortality, transfusion, pericardial complications	- Frailty should be a key factor in procedural decision-making. - Consider alternative strategies (medical therapy, staged PCI, less invasive techniques). - Geriatric and palliative care involvement may be appropriate. - Intensive postprocedural monitoring and tailored care planning essential.

## Data Availability

The National Inpatient Sample database is publicly available at the HCUP-US Home Page (https://hcup-us.ahrq.gov/databases.jsp), accessed on 22 March 2025.

## Supplementary Materials

The following supporting information can be downloaded at: https://www.mdpi.com/article/10.3390/jcm14134745/s1, Table S1: List of ICD-10 codes utilized in the present study, Table S2: Sensitivity analysis including the length of hospital stay in the adjusted models.

## Abbreviations

The following abbreviations are used in this manuscript:

CTO	Chronic total occlusion
PCI	Percutaneous coronary intervention
ICD-10	International Classification of Diseases, Tenth Revision
CI	Confidence interval
OR	Odds ratio
