# Supplementary material for: Frailty as a Predictor of In-Hospital Outcomes in Patients Undergoing Percutaneous Coronary Intervention for Chronic Total Occlusion
Source: J Clin Med. 2025 Jul 4;14(13):4745. doi: 10.3390/jcm14134745 (PMC12250810; doi:10.3390/jcm14134745)
Supplement: Supplementary file 1 [file jcm-14-04745-s001.zip › jcm-3692161-Supplementary.pdf]

SUPPLEMENTARY DATA

Table S1. List of ICD-10 codes

Diagnoses/Procedures	Codes
Chronic total oclussion	I2582
Percutaneous coronary intervention	0270*
Acute myocardial infarction	I21*
Vascular complication	I74*, I75*, I7777, I7776, I7100, I7102, I7101, I7103, S15xA, S25xA, S35xA, S45xA, S55xA, S65xA, S75xA, S85xA, S95xA, S090XXA, T81710A, T81711A, T81718A, T8172XA, I770, I9751, I9752, I97418, I9742, I97618, I9762, I97620, I97621
Blood transfusión	30243N0, 30243N1, 30243P0, 30243P1, 30243H0, 30243H1, 30240N0, 30240N1, 30240P0, 30240P1, 30240H0, 30240H1, 30230H0, 30230N0, 30230N1, 30230P0, 30230P1, 30233N0, 30233N1, 30233P0, 30233P1
Coronary perforation/dissection	I9751, I2542
Pericardial complication	I312, I314
Renal replacement therapy	5A1D70Z, 5A1D80Z, 5A1D90Z

Table S2. Sensitivity analysis including the length of hospital stay in the adjusted models

Outcomes	Adjusted Model*		
	OR	95% CI	*p*-Value
**In-hospital mortality**			
Low risk of frailty		Ref.	
Intermediate risk of frailty	3.50	2.35 - 5.22	<0.001
High risk of frailty	10.2	3.69 - 28.2	<0.001
**Vascular complication**			
Low risk of frailty		Ref.	
Intermediate risk of frailty	1.72	1.23 - 2.43	0.002
High risk of frailty	0.77	0.23 - 2.52	0.663
**Blood transfusion**			
Low risk of frailty		Ref.	
Intermediate risk of frailty	2.42	1.70 - 3.44	<0.001
High risk of frailty	2.07	0.72 - 5.93	0.175
**Coronary perforation/dissection**			
Low risk of frailty		Ref.	
Intermediate risk of frailty	1.23	0.89 - 1.69	0.208
High risk of frailty	0.70	0.22 - 2.28	0.557
**Pericardial complication**			
Low risk of frailty		Ref.	
Intermediate risk of frailty	2.74	1.58 - 4.77	<0.001
High risk of frailty	9.96	3.26 - 30.5	<0.001
**Renal replacement therapy**			
Low risk of frailty		Ref.	
Intermediate risk of frailty	1.84	1.24 - 2.72	0.002
High risk of frailty	3.59	1.18 - 10.9	0.024

* Adjusted for age, sex, race/ethnicity, elective admission, expected insurance payer, bed size of hospital, location of hospital, and length of hospital stay.
